# Supplementary material for: Single-Cell Transcriptomic Landscape of Cervical Cancer Cell Lines Before and After Chemoradiotherapy
Source: Cells. 2026 Jan 8;15(2):115. doi: 10.3390/cells15020115 (PMC12839042; doi:10.3390/cells15020115)
Supplement: Supplementary file 1 [file cells-15-00115-s001.zip › cells-4062518-supplementary.pdf]

Treatment Protocol of AdMer Patient

Both cell lines were derived from a 22 year-old patient after whom the line is named. The patient was diagnosed with stage IIbN1M0 (IIIB) squamous cell carcinoma of the cervix, exophytic form, vaginal-parametrial variant. The disease was diagnosed in June 2023. The patient was treated with three cycles of polychemotherapy according to the scheme paclitaxel combined with a carboplatin, the last course was completed in September 2023. On November 2023, a tumor tissue sample was collected from patient during routine visit in the Academician E.N. Meshalkin National Medical Research Center. The primary culture AdMer35 was subsequently developed from this sample.

In November and December 2023, the patient received concurrent chemoradiation with a single focal dose (SFD) of 2 Gy to a total focal dose (TFD) of 50 Gy supplemented with cisplatin administration.

A repeat course of radiation therapy was conducted in January 2024: 1 session of intracavitary brachytherapy with an SFD of 5 Gy; external boost to the cervical tumor with an SFD of 5 Gy in 4 fractions (TFD 20 Gy), and retroperitoneal lymph node conglomerates with an SFD of 3 Gy in 4 fractions (TFD 20 Gy). Considering the negative dynamics (tumor spread, recurrent massive bleeding from the cervical tumor vessels, and metastasis), gemcitabine therapy was administered in February 2024. Subsequently, another tumor tissue sample was collected in March 2024, resulting in the development of the primary culture AdMer43.

Cell lines AdMer35 and AdMer43 grew attached to the flask, exhibited stable epithelial morphology (cobblestone pattern), adhesion, were positive by cytokeratin staining, and have HPV-59 DNA genomes. AdMer35 cells demonstrated more intensive growth of primary tumor nodes, which is associated with their high motility and migration activity found in vitro and can explain the ability to invade the surrounding tissues more effectively in vivo. In contrast, AdMer43 cells characterized by higher proliferative potential but a relatively low migration rate in vitro, in vivo form smaller, slowly growing tumor nodes as compared to AdMer35. At the same time, AdMer43 demonstrated increased resistance in comparison with AdMer35 to both radiotherapy (minimal apoptosis and G2/M arrest post-5 Gy irradiation) and chemotherapy (IC_{50} for carboplatin: 87.8 μM vs. 54.3 μM; for paclitaxel: 7.3 nM vs. 3.0 nM) [17].

Table S1. RNA markers used to identify senescent-like cells (SnC) with AUCell.

RNA Marker	Description	Ref
CDKN1A (p21)	Cyclin-dependent kinase inhibitor. Functions as a regulator of cell cycle progression at G1	[55]
CDKN2A (p16)	CDK4/6 inhibitor; a classic marker of senescence and aging	[56]
GLB1 (SA-β-gal)	β-galactosidase gene often increased in senescent cells	[57]
IL6	Proinflammatory cytokine - component of SASP	[58]
CXCL8 (IL8)	Chemokine, SASP component, developmental direction and immune cell recruitment	[58]
SERPINE1 (PAI-1)	PAI-1 is a SASP-associated secreted factor that influences senescence induction.	[59]
MMP1 / MMP3	Matrix metalloproteinases are often elevated in SASP; ECM remodeling	[60,61]
CCL2	A chemokine involved in paracrine transmission of senescence and recruitment of immune cells	[62]
IGFBP3	IGF binding protein-3 is a secreted factor associated with TIS and SASP	[63]
GDF15	Stress-responsive cytokine also known as a mitokine often elevated in SASP and with aging	[64]
H2AC19, H2BC10, H2BC13, H2BC26, H2BC5, H2BC9, H3C12, H3C13, H3C15, H4C12, H4C16,	Histone Genes Transcripts	Enricher Library "WikiPathways_2024_Human", "Senescence Associated Secretory Phenotype SASP WP3391"

Table S2. Ligands, receptors and pathways involved in intra- and intercellular interactions identified by CellChat V2 in AdMer35, AdMer43 cells for senescent-like cells (SnC) and stem-like cells (SLC).

SnC	SCL	Pathway	Ligands	Receptors	Ref
Sheared	AdMer43	MK	MDK	SDC2, -4, ITGA4, -A6, -B1, LRP1, NCL, SDC1, -2, -4	[70]
AdMer43	Sheared	EGF	AREG, EREG, HBEGF, TGFA	EGFR, ERBB2	KEGG: hsa04012
AdMer43	Sheared	SEMA3	SEMA3A, -B, -C	NRP1, PLXNA1, -2, -3,-D1	[71]
AdMer43	Sheared	BMP	BMP2	BMPR1A,-R2, -1B, ACVR2A	KEGG:hsa04350; [72]
AdMer43	AdMer43	ncWNT	WNT11	FZD6, -10	KEGG: hsa04310
AdMer35	ND*	CypA	PPIA	BSG	[73]
AdMer35	Sheared	VISFATIN	NAMPT	ITGA5,-B1	[74]
AdMer35	AdMer43	PLAU	PLAU	PLAUR	[75]
ND*	Sheared	GAS	GAS6	AXL	[76]
ND	AdMer43	TGFb	TGFB1, TGFB2	TGFbR1, -R2	KEGG: hsa04350
ND	AdMer43	GRN	GRN	SORT1	[77]
ND	AdMer43	NPW-B	NPW	NPBWR1	KEGG: hsa04080
ND	AdMer43	WNT	WNT7B	FZD6, LRP5, -6	KEGG: hsa04310, [78]
ND	AdMer35	NRG	NRG1	ITGA6, -B4	[79]
ND	AdMer35	SLITRK	SLITRK6	PTPRS	[80,81]

ND* - Not Detected.

Table S3. Molecular markers of stem-like cells (SLC).

Gene	Description	Ref
ABCG2	ATP Binding Cassette Subfamily G Member 2. Highly expressed in stem cells, is associated with stem cell characteristics and aggressive tumor behavior including cervical cancer.	[88–90]
ALDH1A1	Aldehyde Dehydrogenase 1 Family Member A1. Is considered a cancer stem cell marker. Expression of this gene is important for maintaining the viability and differentiation of stem cells, as well as for tumor formation in general.	[91–93]
CD24	CD24. Is a mucin-like cell surface glycoprotein that is frequently overexpressed in various human cancers and is correlated with a poor prognosis. Expression is associated with characteristics of cancer stem cells and linked to metastasis and resistance to chemotherapy in cervical and ovarian cancers.	[89,91,94]
CD44	CD44. Cell-surface glycoprotein involved in cell-cell interactions, cell adhesion and migration. It is a receptor for hyaluronic acid and can also interact with other ligands, such as osteopontin, collagens, and matrix metalloproteinases.	[94,95]
ITGA6	Integrin Subunit Alpha 6 (CD49f). Plays a role in cell adhesion and signaling. Is upregulated in CSCs and can contribute to their stem-like properties, including self-renewal, differentiation, and resistance to therapy	[89,90]
KRT17	Keratin, Type I Cytoskeletal 17. Expression is upregulated in cervical cancer and is associated with stem-like properties like self-renewal	[90,97]
MSI1	Musashi RNA Binding Protein 1. Expression in CSC is associated with poor prognosis in patients with cervical cancer	[89,91]
POU5F1	POU Class 5 Homeobox 1 (OCT3, Oct-3, Oct3/4) is a key regulator of pluripotency in embryonic stem cells and is also expressed in cancer stem cells, which are thought to be responsible for tumor initiation, metastasis, and drug resistance.	[89,91,98]
PROM1	Prominin 1 (CD133). Pentaspan transmembrane glycoprotein. Expression is associated with poor prognosis in various cancers, including cervical cancer.	[90,99]
SOX2	SRY-Box Transcription Factor 2. A common marker of cancer stem cells, including a cervical cancer.	[89–93]

Table S4. Contribution of cervical cancer stem like cells (SLC) to senescent like cell (SnC) subpopulations.

Cell Types	SLC	Non-SLC	SLC (%)	Non-SLC (%)
SnC	190	1318	12.59	9.71
Non-SnC	1319	12258	87.41	90.29
Total	1509	13576	100	100

Table S5. Cell cycle RNA markers used to distinguish cells in S and G2M phases.

Cells Cycle Phase	Transcripts
**S**	MCM5, PCNA, TYMS, FEN1, MCM2, MCM4, RRM1, UNG, GINS2, MCM6, CDCA7, DTL, PRIM1, UHRF1, HELLS, RFC2, RPA2, NASP, RAD51AP1, GMNN, WDR76, SLBP, CCNE2, UBR7, POLD3, MSH2, ATAD2, RAD51, RRM2, CDC45, CDC6, EXO1, TIPIN, DSCC1, BLM, CENPU, MCM10, CDC7, DDX11, RFC4, DONSON, RFC5, CLSPN
**G2M**	HMGB2, CDK1, NUSAP1, UBE2C, BIRC5, TPX2, TOP2A, NDC80, CKS2, NUF2, CKS1B, MKI67, TMPO, CENPF, TACC3, FAM64A, SMC4, CCNB2, CKAP2L, CKAP2, AURKB, BUB1, KIF11, ANP32E, TUBB4B, GTSE1, KIF20B, HJURP, CDCA3, HN1, CDC20, TTK, CDC25C, KIF2C, RANGAP1, NCAPD2, DLGAP5, CDCA2, CDCA8, ECT2, KIF23, HMMR, AURKA, PSRC1, ANLN, LBR, CKAP5, CENPE, CTNNB1

Table S6. The contribution of cells in G1, S and G2M phases of cell cycle in AdMer35 and AdMer43 single cell data was determined using Seurat CellCycleScoring function with a set of RNA markers of cervical cancer stem cells.

Cell Cycle Phase	AdMer43	AdMer35	AdMer43 (%)	AdMer35 (%)
G1	3072	2340	39.97	31.62
S	2220	2596	28.89	35.08
G2M	2393	2464	31.14	33.30
Total	7685	7400	100	100

Table S7. Description of Seurat clusters defined in combined array of cervical cancer AdMer35 and AdMer43 cell lines with characteristics and annotations.

Seurat Cluster	Description	RNA Markers	MSigDB	GO Annotations	Pathways
**0**	**AdMer35** in G1/S phases enriched with SLC	PLA2G7, GRID2, ABCA9, FAT3, LEF1, CHI3L1, IKZF3, MIR646HG, ZFP14, GABRB3, PTPRZ1, HCRTR2, ZNF555	Epithelial Mesenchymal Transition, Hedgehog Signaling, UV Response Dn	Collagen-Containing Extracellular Matrix, Endoplasmic Reticulum Lumen, Chemorepellent Activity	Wnt signaling, Cadherin signaling
**1**	**AdMer43** in G1/S phases	PTPRC, NAP1L3, CYTL1, GBP7, SOX5, MEFV, RARRES1, CHRD, TRPV2, DIO2	Interferon Alpha Response, Interferon Gamma Response, Inflammatory Response, KRAS Signaling Up	Defense Response to Virus, Response to Cytokine, Collagen Containing Extracellular Matrix, Endoplasmic Reticulum Lumen	Cytokine Signaling in Immune System, Extracellular Matrix Organization
**2**	**AdMer43** in G2M phase	H4C4, DMBX1, H2BC14, SAPCD2, MYT1L, IHO1, H2AC14, H3C1, IRX1, H2BC18, H3C10	E2F Targets, G2-M Checkpoint, Mitotic Spindle	Mitotic Sister Chromatid Segregation, Mitotic Spindle	Cell cycle, p53 signaling pathway
**3**	**AdMer35** in G2M phase, enriched with SLC	OR2B6, F13B, OR2B2, PLK1, PIF1, NDC80, FAM72C, H2BC12, FAM83D, DLGAP5, BUB1, CCNA	G2-M Checkpoint,E2F Targets,Mitotic Spindle	Mitotic Sister Chromatid Segregation,Mitotic Spindle Organization,Positive Regulation of Cell Cycle Process	Cell Cycle, Mitotic, Cell Cycle, M Phase
**4**	**Mixed 35/43** in different cell cycle phases, enriched with SLC, high HPV59, and high SnC contribution	SPRR2A, MUC15, KLK9, SPRR1B, TMPRSS4, ALDH3B2, S100A7, PGLYRP3, GJB6, KLK11, MACC1, SCEL	Estrogen Response Early/Late, Apical Junction, KRAS Signaling Up/Dn, p53 Pathway	Apical Junction Complex, Cell-Cell Junction, Keratin Filament	Formation of the Cornified Envelope, Keratinization, Vitamin D Receptor Pathway
**5**	**Mixed 35/43** in different cell cycle phases, enriched with PLC, with low SLC, and high SnC contributions	ACTN2, GADD45G, TEX54, WNT9B, RASAL3, HOXD1, HS3ST2, GRIN1, RAD51AP2, ALKAL1	p53 Pathway, TNF-alpha Signaling via NF-kB, Estrogen Response Early, IL-6/JAK/STAT3 Signaling, DNA Repair	RNA Polymerase II Cis-Regulatory Region Sequence-Specific DNA Binding	p53 pathway Homo sapiens, Senescence Associated Secretory Phenotype
